# Application of reference air kerma alert levels for pediatric fluoroscopic examinations

**DOI:** 10.1002/acm2.13721

**Published:** 2022-08-04

**Authors:** Elanchezhian Somasundaram, Samuel L. Brady, Keith J. Strauss

**Affiliations:** ^1^ Department of Radiology Cincinnati Children's Hospital Medical Center Cincinnati Ohio USA; ^2^ Department of Radiology University of Cincinnati School of Medicine Cincinnati Ohio USA

**Keywords:** alert levels, diagnostic reference levels, entrance skin dose, fluoroscopy, pediatric, radiation dose, reference air kerma

## Abstract

The purpose of this study was to provide an empirical model to develop reference air kerma (RAK) alert levels as a function of patient thickness or age for pediatric fluoroscopy for any institution to use in a Quality Assurance program. RAK and patient thickness were collected for 10&663 general fluoroscopic examinations and 1500 fluoroscopically guided interventions (FGIs). RAK and patient age were collected for 6137 fluoroscopic examinations with mobile‐C‐arms (MC). Coefficients of linear regression fits of logarithmic RAK as a function of patient thickness or age were generated for each fluoroscopy group. Regression fits of RAK for 50%, 90%, and 98% upper prediction levels were used as inputs to derive an empirical formula to estimate alert levels as a function of patient thickness. A methodology is presented to scale results from this study for any patient thickness or age for any institution, for example, the patient thickness dependent RAK alert level at the top 1% of expected RAK can be set using the 98% upper prediction interval boundary given by: ${\mathrm{RAK}}_{98\%}=e^{m.x_{\mathrm{avg}}+s_{98}.\hat{c}}\;$, where *x*_avg_ is the institute's average patient thickness or age, and $\hat{c}$ is the intercept based on the average RAK of the patient population calculated as $\hat{c}=\ln\left({\mathrm{RAK}}_{\mathrm{avg}}\right)\;-m.x_{\mathrm{avg}}.\mathrm{RA}K_{\mathrm{avg}}$ is the institution's average RAK (mGy). *m* and *s*_98_ are constants presented for each type of fluoroscope and RAK group and represent slope of the fit and scale factor, respectively. An empirical equation, which estimates alert levels expressed as air Kerma without backscatter at the interventional reference point as a function of patient thickness or age is provided for each fluoroscopic examination type. The empirical equations allow any facility with limited data to scale the results of this study's single facility data to model their practice's unique RAK alert levels and patient population demographics to establish pediatric alert levels for fluoroscopic procedures.

## 1 INTRODUCTION

To establish a robust quality assurance (QA) program in fluoroscopy, an institution should define^1^ alarm levels, which are radiation exposure notification thresholds based on potential skin effects (i.e., epilation and erythema) and^2^ alert levels, which are notification thresholds based on examination‐specific radiation exposures that exceed a reasonable level for a specific patient demographic (such as body thickness or age).

With respect to alarm levels, adult fluoroscopically guided interventions (FGIs), due to their complexity and subsequent elevated radiation doses, are more likely to cause skin injuries than other fluoroscopic examinations. In the early 1990s, the United States Food and Drug Administration began receiving reports of substantial skin injuries due to FGIs.^1^ By 2010, the radiation effects on patients’ skin and hair, and the required threshold radiation doses were better understood and documented.^2^, ^3^ Radiation exposure alarm levels for adults, based on peak skin dose, reference point air Kerma, Kerma‐area product, or fluoroscopy time during FGI were developed for adults.^4^, ^5^ In addition, post procedure thresholds^6^ were established to identify patients that would require follow‐up to properly manage potential skin effects.^4^

With respect to alert levels, radiation dose from all types of fluoroscopic procedures, that is, general fluoroscopy, mobile C‐arm fluoroscopy (typically employed in a surgery setting), and most FGIs, do not exceed alarm levels that would cause a skin effect, but may be inappropriately high with respect to other similarly performed procedures within a specific patient demographic (i.e., based on patient body thickness or patient age). In addition to alarm levels set for high‐level, complex FGI procedures, a robust fluoroscopic QA program will identify appropriate alert levels for all different fluoroscopic procedures to help mitigate unnecessarily elevated patient doses. These elevated doses can be due to a malfunctioning fluoroscope, ineffective management of the fluoroscope's controls by the operator, or incorrect configuration of the fluoroscope to address unique requirements of the fluoroscopic examination such as fluoroscopically imaging a 3 kg infant as opposed to a 150 kg adult.^7^ Furthermore, a QA program that helps manage the patient dose for an institution must be adjustable to account for the site's unique patient population, especially if pediatric patients are involved. The AAPM's recently published Practice Guideline 12.a^8^ is an excellent guide with respect to establishing alarm levels for adult fluoroscopy. However, its scope did not include the importance of establishing alert levels as a function of patient size, which is an important task within a QA program for fluoroscopy of both pediatric and small sized adult patients.

This study at a large tertiary care pediatric hospital uses pediatric reference air kerma (RAK) data from general fluoroscopic examinations and FGIs within Radiology, and from MC fluoroscopes in the operating room to develop pediatric fluoroscopy QA RAK alert levels as a function of patient thickness and or age. An algorithm is developed to allow adjustments of this study's results to estimate RAK alert levels at other institutions or for a given institution to periodically update RAK alert levels when new technology or methodology is implemented that has changed patient dose levels.

## 2 METHODS

### 2.1 Data collection

This retrospective study anonymized all patient data and was compliant with the Health Insurance Portability and Accountability Act. The Institutional Review Board waived the need for consent for this QA study. The lateral and anteroposterior thickness of the majority of patients imaged with ionizing radiation were measured using body calipers. Patient body thickness was the preferred correlation metric to be used to characterize RAK for alert levels since the thickness of the abdomen of the largest 3‐year‐old has been shown to be the same as the smallest 18‐year‐old.^9^ However, sterile draping of the patient within the operating room prohibited thickness measurements with calipers. Thus, patients in the operating room were grouped by the less preferrable method of age. All measured patient thicknesses, ages, and the DICOM Radiation Dose Structured Report (RDSR) data provided by each fluoroscope were recorded in a dose database (Clinical Microsystems Corporation, Riva, MD, USA) from 10/2016 through 12/2019. All examinations with complete data in the database, within the period of the study, were included.

### 2.2 Procedure groupings

Patient data were queried and grouped for three common types of fluoroscopes: general fluoroscopes with tilting table (GF), single or biplane interventional fluoroscopes designed for FGI examinations, and mobile C‐arms in the operating room (MC). For each type of fluoroscope, patients were further divided into groups based on the procedure's complexity and imaged body region to reduce the range of doses within each group.^10^ For the GF fluoroscope, the four most common types of pediatric GF examinations were created in individual groups. For MC and FGI examinations, different types of examinations with similar expected complexity were grouped together to create low, medium, and high RAK groups. Total fluoroscopy time of the examination and expert clinical judgment was used to estimate the complexity of each type of examination. All patients undergoing a specific examination type were assigned to the same RAK group. Basic specifications of each fluoroscope are provided in Table 1. Table 2 lists the examination descriptions grouped together to create 10 patient groupings along with the median age and median weight.

**TABLE 1:** Basic specifications and quantity of each fluoroscope used in study

Type	Quantity	Manufacturer	Model	Reference point* (cm)
MC	3	Philips Healthcare Solutions Amsterdam, Netherlands	Veradius Unity Surgical C‐arms	70 cm
MC	7	General Electric Healthcare Boston, MA, USA	OEC Surgical C‐arms: Elite, 9900 Smartview, and 9800	70 cm
GF	5	Philips Healthcare Solutions Amsterdam, Netherlands	Easy Diagnost, undertable x‐ray tube mounted on tilt table	65 cm
FGI	1	Philips Healthcare Solutions Amsterdam, Netherlands	Allura Xper FD20 with Clarity Image Processing: Single Plane	65 cm
FGI	1	Philips Healthcare Solutions Amsterdam, Netherlands	Azurion with FD20 with Clarity Image Processing: Single Plane	65 cm
FGI	1	Philips Healthcare Solutions Amsterdam, Netherlands	Azurion with FD 20 with Clarity Image Processing: Biplane Unit	65 cm

Abbreviations: FGI, fluoroscopic guided intervention examination; GF, general fluoroscopic examination in Radiology; MC, mobile C‐arm fluoroscopic examination in Operating Room.

*Reference Point is distance from the focal spot towards the image receptor.

**TABLE 2:** Ten patient groupings based on machine type, dose level, and examination types

Unit type	Groups	Exam description	Median age (IQR) in Years	Median weight (IQR) in kg	Median RAK(IQR) in mGy
General fluoroscopy (GF)	Abdomen	GastroIntestinal (GI)	4.77 (0.60–12.12)	14.90 (5.78–37.00)	1.52 (0.76–3.86)
	Abdomen/Pelvis	Voiding cystourethrogram (VCUG)	3.33 (0.575–8.08)	12.97 (5.79–24.60)	0.36 (0.16–0.92)
	Lateral Head and Neck	Video swallow study (VSS)	2.30 (0.63–8.32)	11.58 (6.61–23.7)	0.71 (0.41–1.39)
	Trunk	Tube placement (TP)	4.80 (1.02–13.17)	15.50 (8.38–33.20)	2.35 (1.53–3.54)
Mobile C‐ arm in operating room (MC)	Low RAK	Distal extremities including knee or elbow	11.44 (6.64–14.87)	Not recorded	1.06 (0.31–2.81)
	Medium RAK	Proximal extremities before knee or elbow spine, scoliosis, dilations, cystography, shoulders hips, tube placement and exchange, ureteroscopy	10.47 (4.12–15.82)	Not recorded	2.72 (0.95–7.13)
	High RAK	Endoscopy	9.96 (4.52–15.14)	Not recorded	9.64 (2.07–26.44)
Fluoroscopic‐guided intervention (FGI)	Low RAK	Pic line placement/exchange direct sclerosing drains arthrograms steroid injections	12.40 (5.38–16.72)	42.30 (17.30–64.78)	2.00 (1.20–4.93)
	Medium RAK	Catheter change/drain CBCT‐guided needle placement dilation esophagus fluoro guidance local diag/thera‐spine fluoro‐IR ERCP Perc place IVC filtration	11.71 (3.62–16.91)	42.20 (16.10–66.30)	9.3 (3.30–28.60)
	High RAK	Carotid Angiograms Cholangiograms Diagnostic Cerebral Angiogram Visceral Angiograms Embolizations Venography	12.77 (3.7–17.31)	39.60 (15.20–64.30)	108.90 (41.25–296.25)

RAK: air Kerma without backscatter at interventional reference point.

IQR: Interquartile range.

The dose index captured in the RDSR was the, air Kerma without backscatter^11^ at the interventional reference point^12^ from the focal spot of the fluoroscope (K_a,r_); hereafter RAK. Calibration correction factors, calculated from radiation output measurements conducted annually during compliance testing of each fluoroscope,^13^ were applied to display cumulative RAK values in the stored RDSRs to reduce error in the reported RAK values to be better than ± 5%. RAK is the chosen dose index to be applied for alert levels instead of the use of Kerma Area Product (KAP) since radiation levels are a primary concern with respect to alert levels. Additionally, the use of RAK avoids introducing a second form of error in the analysis, namely: collimator blade motion accuracy (i.e., actual collimated field size vs. displayed collimated size used to calculate KAP).

### 2.3 RAK fits based on patient thickness and age

The normality of the RAK data for each procedure group was assessed visually using quantile‐quantile plots^14^; logarithmic transformation was used to transform the data into a normal distribution. Quantile–quantile plots and residual plots of the transformed data were used to verify the normality and homoscedasticity assumptions of the data for linear regression.

The log‐transformed RAK data were fitted as a function of patient thickness measured in the anteroposterior or lateral dimension based on the procedure for all fixed modalities using a linear regression model, such that the logarithmic RAK ($\hat{y}$) was given by:

(1) $$\;\hat{y}=\;bx+c,\;$$

where *b* was the slope, *x* is the patient thickness (in centimeters), and *c* was the intercept. The linear model predicts the mean logarithmic RAK values. For mobile C‐arm procedures, age (in years) was substituted for patient thickness. To establish dose alert levels, the upper limit of the prediction interval for the linear fit was calculated. The prediction interval^15^ provided a spread for the expected dose observations for a new patient of thickness (or age) *x*_new_ and under the large sample assumption, can be determined using the equation:

(2) $$R\to \;{\hat{y}}_{\mathrm{new}}\pm Z_{1-\alpha }\;S_{\mathrm{pred}}\left(x_{\mathrm{new}}\right),$$

where ${\hat{y}}_{\mathrm{new}}$ was the expected mean dose for the new patient calculated using equation Eq.1, $Z_{1-\alpha }$ was the z‐score from normal distribution, and α was the level, which determines the width of the prediction interval. As an example, to set the dose alert at the upper 5% level of the prediction interval, α was set to 0.05. Finally, *S*_pred_($x_{\mathrm{new}})\;$was the error term for the prediction interval for a patient of thickness (or age) *x*_new_ and was defined as,

(3) $$S_{\mathrm{pred}}\left(x_{\mathrm{new}}\right)=\sqrt{s^{2}\left(1+\frac{1}{N}+\frac{{\left(x_{\mathrm{new}}-\bar{x\;}\right)}^{2}}{\sum_{i=1}^{N}{\left(x_{i}-\bar{x}\right)}^{2}}\right)},$$

where *s* ^2^ was the mean‐squared error of the linear regression fit, $\bar{x}$ was the average patient thickness (or age) in the dataset, and *N* is the number of examinations in the group. From Equations 1–3, the dose alert level ($D_{\alpha })$ for a given patient thickness *x*_new_ and α‐level was determined as:

(4) $$\begin{array}{ccc} & & RAK_{\alpha }\;\left(x_{\mathrm{new}}\right)\\ & & \;=\;EXP\left(bx_{\mathrm{new}}+c\;+\;Z_{1-\alpha }\sqrt{s^{2}\left(1+\;\frac{1}{N}+\frac{{\left(\;x_{\mathrm{new}}-\bar{x\;}\right)}^{2}}{\sum_{i=1}^{N}{\left(\;x_{i}-\bar{x}\;\right)}^{2}}\right)}\;\right). \end{array}$$

In equation Equation 4, the exponential (EXP) operation was applied to inverse the log transform and yield the RAK in units of mGy for setting the alert levels.

For clinical application, the prediction interval upper boundaries (from Equation 4) for three different α values (for 50%, 90%, and 98% levels), as a function of patient thickness ranging from 0 to 40 centimeters, and as a function of patient age ranging from 0 to 21 years, were plotted, and linear regression fits of these plots were calculated. The 50% upper prediction boundary estimated the level above which 25% of the expected RAK occurred for this study's population. Similarly, the 90% and 98% upper prediction boundaries corresponded to the levels above which 5% and 1% of the expected RAK occurred. The coefficients of the linear fits of these levels were used to set desired alert level $\hat{\mathrm{RAK}}$ defined in Equation 5:

(5) $${\hat{\mathrm{RAK}}}_{\alpha }\left(x_{\mathrm{new}}\right)=\mathrm{EXP}\left(m\cdot x_{\mathrm{new}}+s_{\alpha }\cdot \hat{c}\right),$$

where $\hat{c}$ is the intercept value of the linear dose fit and may be calculated for each institution based on the average *RAK*_avg_ for their average size or age patient (*x*_avg_) as:

(6) $$\;\hat{c}=\ln\left({\mathrm{RAK}}_{\mathrm{avg}}\right)\;-m\cdot x_{\mathrm{avg}},\;$$

where *m* was the mean slope of the upper prediction boundary, and $s_{\alpha }$ was the ratio of the intercepts for the dose fit to the upper prediction boundary fit. *m* and $s_{\alpha }$ are predetermined constants provided for each modality and dose group.

The slopes of the fits for each group were compared by fitting a single anova model with thickness (or age) as the co‐variate and group as the treatment variable. A post hoc Tukey's test was performed using the emmeans package^16^ in R to determine the groups that had significantly different (*p* < 0.05) slopes.

### 2.4 Application

Data collected in this study were used to create an empirical model to calculate a RAK alert level for a specific fluoroscopic procedure, summarized by Equations 5 and 6. Equation 5 allowed the calculation of alert level RAKs for any patient thickness (or age) using this study's single institution data. To account for differences in RAK, between this study's results and those at other institutions, Equation 6 enables calculation of $\hat{c}$. When $\hat{c}$ for a different institution is substituted into Equation 5, the resulting alert level RAK will be scaled to account for the unique RAKs at that institution assuming that institution can quantify the average RAK for a sample of patients of a particular body thickness or patient age for a specific fluoroscopic procedure.

## 3 RESULTS

**FIGURE 1:**
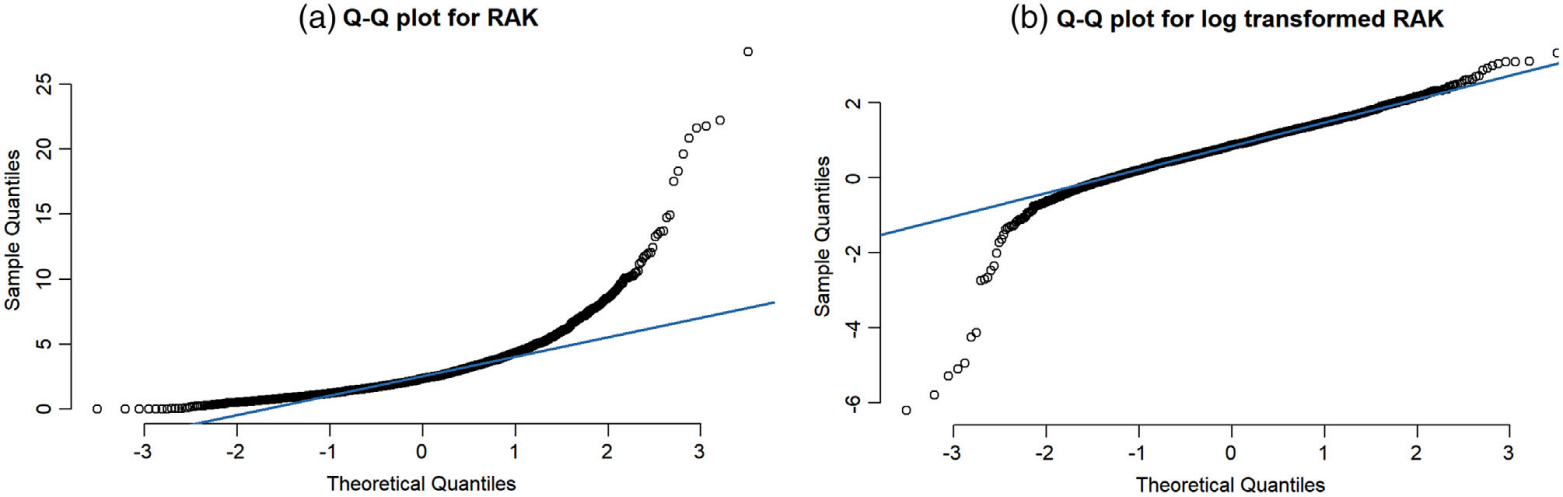
Reference air kerma (RAK) quantile–quantile (Q–Q) plots. Residual plots using the (a) linear regression fits of normal dose in mGy and (b) log transformed dose for the voiding cystourethrogram (VCUG) group in general fluoroscopy

Total numbers of patient examinations included in this study were 10 663—GF, 1500—FGI, and 6137—MC. For each of the 10 patient groupings, Table 2 lists the median age, weight, and RAK with interquartile range. The four GF groups are studies commonly performed on smaller children; the median age is less than 11 years. The median ages for FGI and MC studies ranged from 11 to 12 years. Figure 1 shows the diagnostic plots for VCUG group in general fluoroscopy for linear regression fits that are generated before and after log transformation of the dose data. The quantile–quantile plots in Figure 1a,b show that the normality assumption is met by applying log transformation to the RAK values.

**FIGURE 2:**
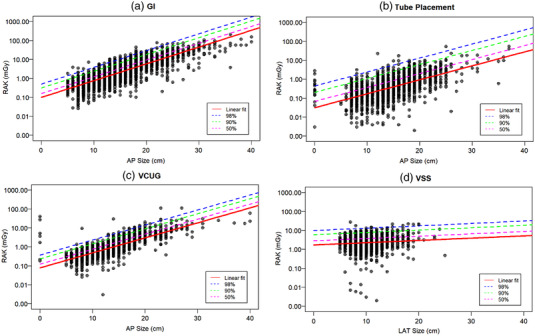
Log reference air kerma (RAK) as function of patient thickness for general fluoroscopy (GF) procedure groups: (a) gastro‐intestinal (GI), (b) tube placement (TP), (c) voiding cystourethrogram (VCUG), and (d) video swallow study (VSS). The reference air kerma (RAK) data were fit for 50%, 90%, and 98% upper prediction levels in addition to a linear fit

**FIGURE 3:**
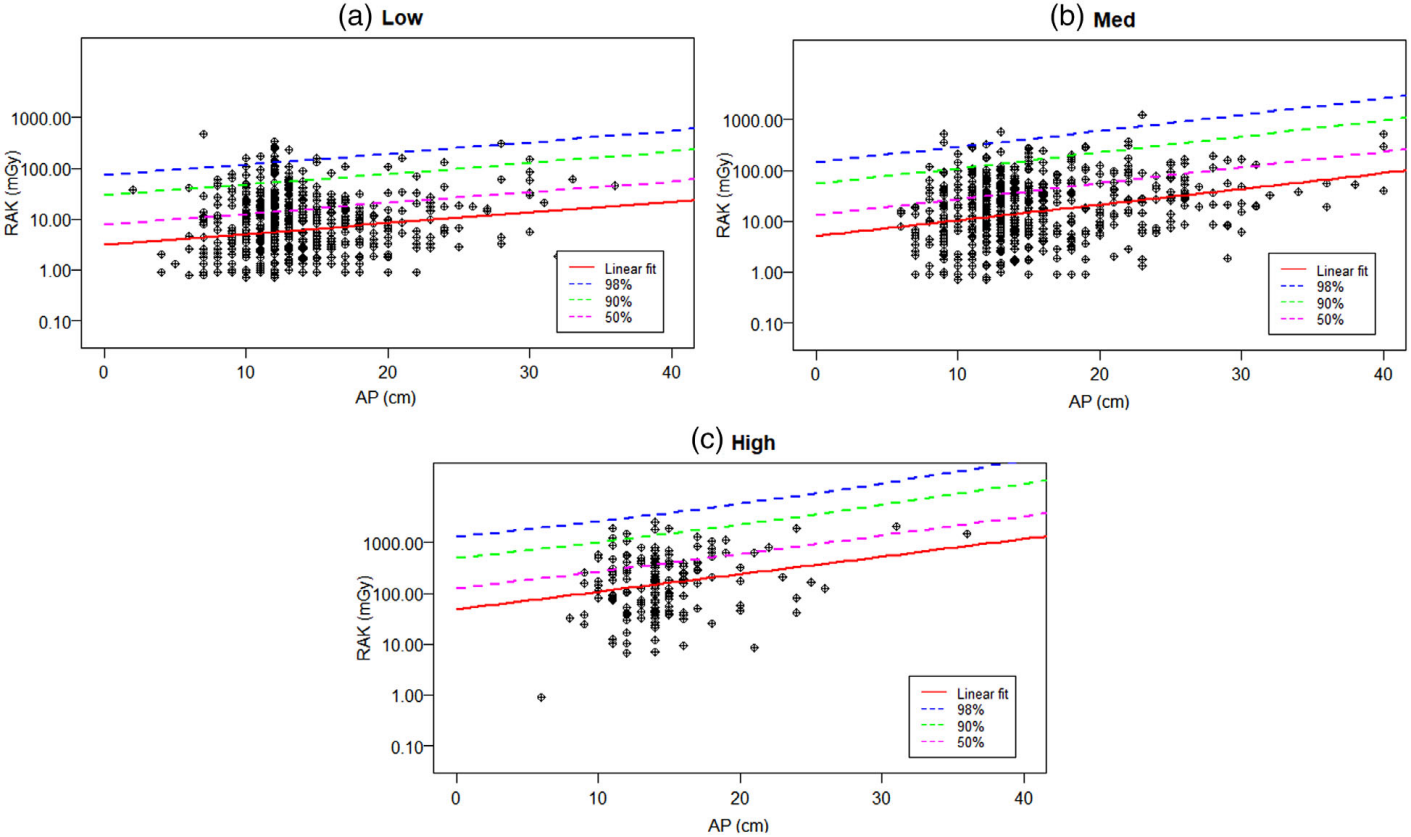
Log reference air kerma (RAK) as function of patient thickness for fluoroscopy‐guided interventional (FGI) procedures groups: (a) studies with low reference air kerma (RAK) values (Low), (b) studies with medium RAK values (medium), and (c) Studies with high RAK values (High). The reference air kerma (RAK) data were fit for 50%, 90%, and 98% upper prediction levels in addition to a linear fit

**FIGURE 4:**
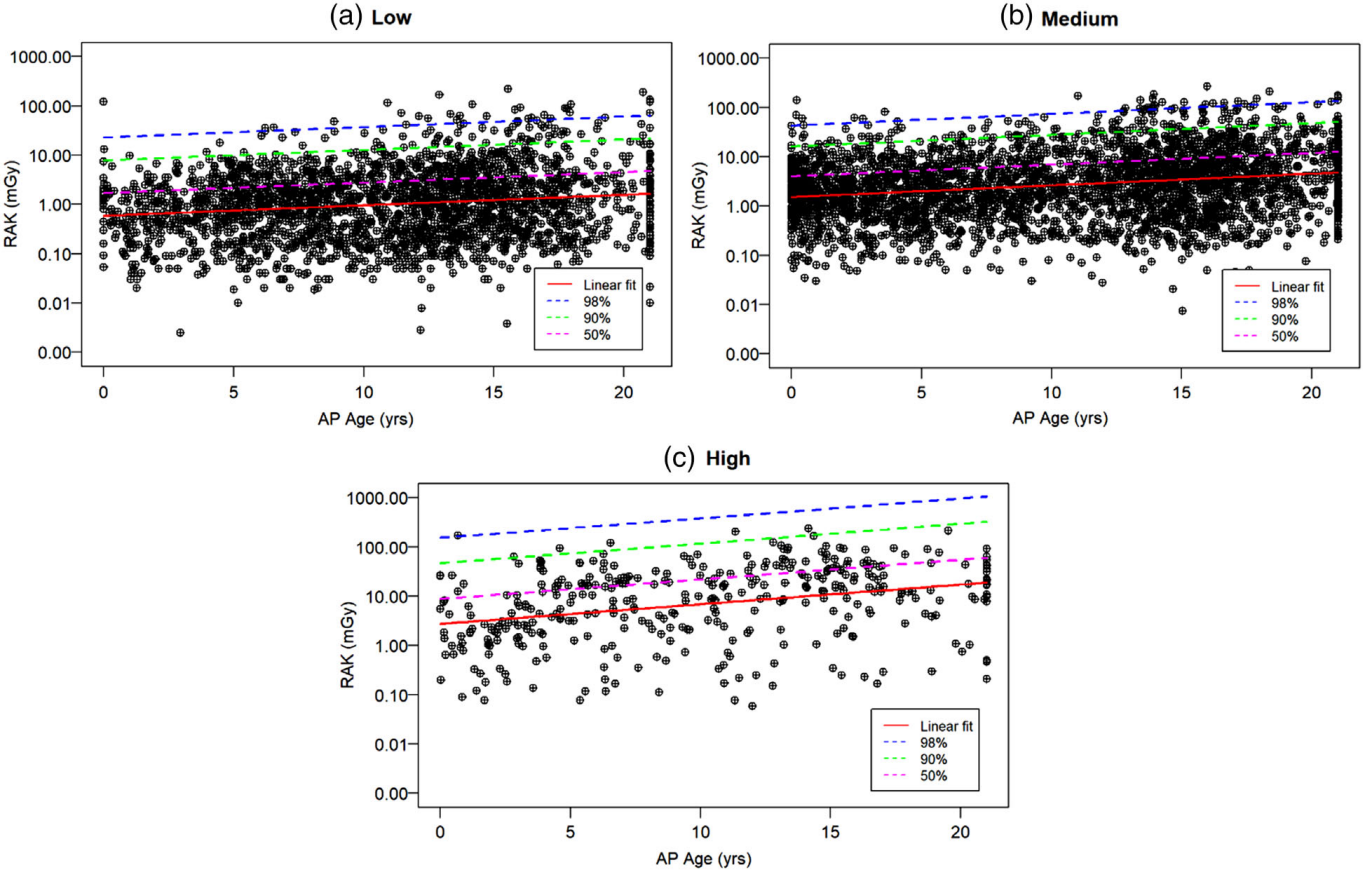
Log reference air kerma (RAK) as function of patient age for mobile C‐arm (MC) groups: (a) studies with RAK values (Low), (b) studies with medium RAK values (medium), and (c) studies with high RAK values (High). The RAK data were fit for 50%, 90%, and 98% upper prediction levels in addition to a linear fit

Figures 2–4 show, for a given type of examination, the linear fit of the log‐transformed RAK as a function of patient thickness (or age), which allows the calculation of mean RAK values using Equation 1. The upper prediction intervals at 50%, 90% and 98% levels are also fitted, which serve as different dose alert levels. Figure 2 shows the plots within GF; the sample sizes for GI, TP, VCUG, and VSS are 4513, 1846, 2070, and 2234, respectively. Figure 3 plots RAK for FGIs with sample sizes in each group of 166, 640 and 694 for high, medium, and low complexity (expected dose and fluoroscopy time) FGI examinations, respectively. The plots for MC are shown in figure 4 with patient ages of 0–21 years for the abscissa instead of thickness. The number of studies for the low, medium, and high RAK groups are 2227, 3544, and 366, respectively.

Table 3 shows the coefficients for Equation 5, the *RAK*_avg_ for the average sized patient, and *RAK*_avg_ for a 23 cm adult in this study, for each of the procedure groups. This allows a different institution to use Equations 5 and 6 to calculate alert level RAK estimates for types of fluoroscopes and examinations similar to those of this study for pediatric patient sizes that are not performed routinely.

**TABLE 3:** Coefficients for calculating RAK levels for each patient group

Group	Slope (m)	S_50_(50% level)	S_90_(90% level)	S_98_(98% level)	Intercept $\hat{c}$	*RAK_avg_* (mGy)	Average thickness (cm)	Adult *RAK_avg_* (mGy) @ 23 cm
**General fluoroscopy**								
GI	0.204	0.798	0.506	0.301	‐2.29	1.76	14.0	10.92
TP	0.171	0.782	0.468	0.248	‐3.45	0.37	14.5	1.57
VCUG	0.183	0.826	0.575	0.398	‐2.54	0.83	12.9	5.21
VSS	0.030	1.898	3.190	4.098	0.54	2.25	9.8	3.24
**Fluoroscopically guided interventions**								

Group	Slope (m)	S_50_(50% level)	S_90_(90% level)	S_98_(98% level)	Intercept $\hat{c}$	*RAK_avg_* (mGy)	Average thickness (cm)	Adult *RAK_avg_* (mGy) @ 23 cm
Low	0.052	1.75	2.84	3.60	1.15	6.03	13.4	9.6
Medium	0.074	1.57	2.40	2.98	1.63	14.8	14.9	26.4
High	0.096	1.19	1.47	1.67	3.88	154	14.5	306
**Mobile C‐arms**								

Group RAK	Slope (m)	S_50_ (50% level)	S_90_ (90% level)	S_98_ (98% level)	Intercept $\hat{c}$	*RAK_avg_* (mGy)	Average Age (Years)	Adult *RAK_avg_* (mGy) @ 21‐year‐old
Low	0.049	‐0.975	‐3.817	‐5.815	‐0.54	0.99	10.8	1.64
Medium	0.055	3.336	6.697	9.059	0.41	2.64	10.2	4.76
High	0.092	2.147	3.802	4.970	1.01	6.87	10.0	18.86

Abbreviations: Abdomen + MSK, abdominal or musculoskeletal system; Angio + Neuro, body or head angiograms; Pic + Scleral, Pick line or Scleral Studies; GI, Gastrointestinal; S50, 50% upper prediction level; S90, 90% upper prediction level; S98, 98% upper prediction level; RAK: air Kerma without backscatter at interventional reference point; TP, Tube Placement; VCUG, Voiding Cystourethrogram; VSS, Video Swallow Study.

The post hoc test comparing the slopes of the different groups of fluoroscopy examinations showed no statistically significant difference for TP and VCUG procedures within GF (*p*‐value = 0.09). VSS and GI slopes were statistically different because VSS procedures were typically performed at higher dose and framerates, and GI procedures were not as straight forward, procedurally, as TP and VCUG procedures and have slightly different dose output requirements than TP, VCUG, and VSS. Additionally, the low and medium groups within MC (*p*‐value = 0.71) and the three FGI groups (*p*‐values ‐ ABM‐AN: 0.54, ABM‐PS: 0.22, AN‐PS: 0.10) did not show statistically significantly different slopes from each other. The relatively smaller sample sizes for FGI studies, especially for PS (*n* = 126), may have attributed to this.

## 4 DISCUSSION

This study empirically models the increase in air kerma without backscatter (RAK) at the interventional reference point as a function of the thickness of the body region irradiated during fluoroscopic examinations with either tilt table units or FGIs within Radiology. A similar model that substitutes age for preferable thickness data with mobile fluoroscopic C‐arms in the operating room is provided. Four empirical fits for each grouping of examination as a function of patient thickness or age are provided. The average linear fit of this study's data is provided to allow any facility to scale the results of this study's single facility data to estimate and model their practice's unique RAK estimates. Assuming our large pediatric population is representative of the typical age and size distribution within the larger pediatric population of the country, the RAK alert levels, as a function of patient size or age, derived using this work should be a reasonable approximation for other institutions.

The RAK values greater than the 90th or 98th upper prediction levels identify examinations from all patient sizes that should/could be investigated as part of a QA Program to ensure good patient care. The RAK of an infant that exceeds the 98th upper prediction level typically is more than an order of magnitude smaller than the 98th upper prediction level for an adult patient. While this infant's RAK probably is substantially lower than an exposure event that would lead to a skin effect, this examination should be reviewed to identify and rectify the cause of the relative (to the patient population) high dose to help prevent similar re‐occurrences during future examinations.

The empirical model of this study requires scaling to represent RAK values of a different facility by adjusting this model's intercept values. This is required to adjust for differences of makes and models of fluoroscopic units, different x‐ray beam filtration, voltages, tube currents, pulse widths, pulse rates, air Kerma at the image receptor (dependent on tolerance of radiologists of image noise), geometry of the fluoroscopes (varying source to skin distance), familiarity and skill level of operators with their fluoroscopes, complexity of examinations, and configuration of the fluoroscope (by the manufacturer in consultation with the staff at the facility to manage the unique imaging requirements of each facility's fluoroscopic examinations).^7^, ^17^

A sample calculation illustrates the application of the empirical model of this study to model RAK alert levels for a different facility. These calculations can be set up by the facility's qualified medical physicist who performs annual compliance testing on the fluoroscopes. Assume an adult facility calculates the average RAK for a sampling of adult patients with 25‐cm anteroposterior thickness to be 20 mGy for GI studies. The unique intercept value for the adult facility, which appropriately scales the empirical model of this study, is calculated with Equation 6 and coefficient data from Table 3:

$$\begin{array}{ccc} \hat{c} & = & \ln\left({\mathrm{RAK}}_{\mathrm{avg}}\right)-m\cdot x_{\mathrm{avg}}=\ln\left(20\right)-0.204\cdot 25=-2.104 \end{array}$$

The RAK alert level based on the 98th upper prediction level can then be calculated for a 2‐year‐old toddler with a 12‐cm thick anteroposterior projection and for the adult patient with a 25‐cm anteroposterior thickness using Equation 5 and coefficient data from Table 3:

$${\hat{\mathrm{RAK}}}_{\alpha }\left(x_{\mathrm{new}}\right)=\mathrm{EXP}\left(m\cdot x_{\mathrm{new}}\;+\;s_{\alpha }\cdot \hat{c}\right)$$

$$\begin{array}{ccc} {\mathrm{RAK}}_{12\mathrm{cm}} & = & \mathrm{EXP}\left(0.204\cdot 12\;+\;0.301\cdot -2.104\right)\\ & = & \mathrm{EXP}\left(1.815\right)=6.1\;mGy\;\mathrm{alert}\;\mathrm{level}. \end{array}$$

$$\begin{array}{ccc} {\mathrm{RAK}}_{25\mathrm{cm}} & = & \mathrm{EXP}\;\left(0.204\cdot 25\;+\;0.301\cdot -2.104\right)\\ & = & \mathrm{Exp}\;\left(4.467\right)=\;87\;mGy\;\mathrm{alert}\;\mathrm{level}. \end{array}$$

If a facility does not have patient thickness data, it can estimate the average size of a patient based on the patient's age using published data for head, thorax, abdomen, or pelvis.^9^ Ideally, the patient thickness should be measured at the time of the fluoroscopic procedure with calipers made available in a radiology department.

A few studies in the United States of pediatric patient dose levels during fluoroscopy from singe institutions have been published.^18^, ^19^, ^20^, ^21^, ^22^ While dose data as a function of patient thickness are limited, two comparisons can be made. A publication in 2008 found that for 21 patients 10–14 cm in thickness received an average RAK of 0.58 mGy with a (0.28–0.80) 95% confidence interval for a VCUG examination.^20^ In this study, the corresponding empirical fit resulted in a RAK of 0.65 mGy, which is in reasonable agreement. Another publication in 2015^18^ lists a RAK of 12 mGy for 175 patients, average age 11.6 years, receiving a variety of FGI examinations of the trunk or peripherals. In this study, the corresponding empirical fit resulted in a RAK of 13 mGy, which is similarly in reasonable agreement.

While this is not a study of DRLs, a comparison of our model's RAK to recently published DRLs can be used for validation of this study's results. The 50% upper prediction boundary of this study's model, which estimates the level above which 25% of the expected RAK occurred for our institution's population, represents values corresponding to a DRL level.^20^ This study's RAK values are converted to KAP, using an average area of the X‐ray beam at the source to skin distance of the patient of 120 cm^2^.^18^ The difference between this study's RAK values converted to KAP DRLs and data published in 2019 from the United Kingdom ranged from 44% to 83%, 57% to168%, and 27% to 106% as a function of patient thickness for VCUG, GI, and VSS examinations respectively with GF fluoroscopes.^23^ This study's results compared to similar data published in 2010 ranged from 8% to 62%, 13% to 123%, and 44% to 55%, respectively.^24^ A study from 36 European countries published in 2014 ranged from 15% to 25% for ages 1–14 years for VCUG examinations.^25^ This study's results are less than values from published results in previous years, as expected.^10^ ^.^ The range of measured RAK values for a single patient thickness in our study is larger, as expected,^9^ than previous published DRLs, which typically report a single average RAK for a group of patients comprised of a small range of ages. The difference between this study's RAK values converted to KAP DRLs for adult sized FGI DRL data published in 2021 in Europe for percutaneous transhepatic biliary drainage, cerebral angiography,^26^ or vertebroplasty^27^ examinations was 112%, 64%, or 34%, respectively. Finally, this study's KAP DRL estimates for adult foot/ankle, elbow, and hip orthopedic MC fluoroscopies in the operating room compared to European published DRLs published in 2019 were 99%, 75%, and 66%,^28^ respectively.

This study has some limitations. The limited number of patients for some of the specific examination types within FGI or MC required combining multiple types of examinations (with similar complexities) within the same group to create larger sample sizes for each fitted equation. Total fluoroscopy time of the examination and expert clinical judgement was used to estimate the complexity of each type of examination type as opposed to statistical groupings. Since all the RAK data come from one institution, these data, by itself, cannot be the basis for pediatric DRLs or universal RAK alert levels. While patient thickness as opposed to age is the preferred model for correlation to RAK, age was the only available parameter as an indication of patient size during sterile procedures in the operating room. While peak skin dose or other patient dose indices are more directly related to risk to the patient than RAK data,^29^ RAK is a fundamental starting point from which a qualified medical physicist can estimate desired patient doses with appropriate calculations.

## 5 CONCLUSION

Data presented in this study allow the estimation of RAK alert levels for most types of pediatric fluoroscopic examinations, except cardiology, from the smallest to largest pediatric patient. Alert levels for all pediatric fluoroscopic examinations in radiology or the operating room will be substantially less than alarm levels used for adult FGIs to monitor for skin effects. For all fluoroscopic examinations, RAK alert levels for pediatric patients should be adjusted for the patient's thickness; for the smallest compared to the largest pediatric patients, the reduction is typically more than an order of magnitude. Pediatric fluoroscopic alert levels based on patient thickness should be used within a QA program to verify appropriate patient care during pediatric fluoroscopic examinations.

## CONFLICT OF INTEREST

The authors declare that there is no conflict of interest that could be perceived as prejudicing the impartiality of the research reported.

## AUTHOR CONTRIBUTION

Elanchezhian Somasundaram, Samuel L. Brady, and Keith J. Strauss have made substantial contributions to the conception and design of the manuscript, actively acquired, analyzed, and interpreted data of the manuscript, drafted and/or revised manuscript for intellectual content, approved the final submitted manuscript, and agree to be accountable for all aspects of the manuscript.
