# Application of reference air kerma alert levels for pediatric fluoroscopic examinations

**DOI:** 10.1002/acm2.14398

**Published:** 2024-08-06

**Current Authors**:

Elanchezhian Somasundaram, Samuel Loren Brady, and Keith Jerel Strauss

Department of Radiology, Cincinnati Children's Hospital Medical Center, Cincinnati OH

Department of Radiology, University of Cincinnati School of Medicine, Cincinnati, OH

**Institution of Origin**:

Cincinnati Children's Hospital Medical Center

MLC 5031, 3333 Burnet Avenue

Cincinnati, OH 45229‐3026

**Corresponding Author**:

Samuel L. Brady, MS PhD, FAAPM

Department of Radiology

Cincinnati Children's Hospital Medical Center

MLC 5031, 3333 Burnet Avenue

Cincinnati, OH 45229‐3026

samuel.brady@cchmc.org

**Original Citation**

Somasundaram E, Brady SL, Strauss KJ, Application of reference air kerma alert levels for pediatric fluoroscopic examinations J Appl Clin Med Phys. 2022;e13721.

**Author Original Contribution Statement**:

All three authors have:

- Made substantial contributions to the conception and design of the manuscript,
- Actively acquired, analyzed, and interpreted data of the manuscript,
- Drafted and/or revised manuscript for intellectual content,
- Approved the final submitted manuscript, and
- Agree to be accountable for all aspects of the manuscript.

**Disclosures**:

No COI for E.S., S.L.B., nor K.J.S. No funding supported this project.

ERRATUM: Application of reference air kerma alert levels for pediatric fluoroscopic examinations

**Equation (** **5** **) had a typographical error; the equation should read**:

(5) $${\hat{RAK}}_{\alpha }\left(x_{new}\right)=EXP\left(m\cdot x_{new}+s_{\alpha }+\hat{c}\right),$$

where, $\hat{c}$ is the intercept value of the linear dose fit and may be calculated for each institution based on the average *RAK_avg_* for their average size or age patient ($x_{avg})$, *m* was the mean slope of the upper prediction boundary, and $s_{\alpha }$ was the logarithmic ratio of the dose at trigger level α to the mean dose level at a given patient size or age x. **Coefficients** $s_{\alpha }$ **were updated in** **Table** **3** **based on the newly modified Equation (** **5** **). A sample calculation is provided to illustrate the application of the empirical model in our previous study with the updated Equation (** **5** **)**. The calculation illustrates the application of the empirical model of this study to model RAK alert levels for a different facility. Assume an adult facility calculates the average RAK for a sampling of adult patients with 25 cm anteroposterior thickness to be 20 mGy for GI studies. The unique intercept value for the adult facility, which appropriately scales the empirical model of this study, is calculated with Equation (6) and coefficient data from Table 3:

$$\hat{c}=\ln\left(RAK_{avg}\right)-m\cdot x_{avg}=\ln\left(20\right)-0.204\cdot 25=-2.104$$

**TABLE 3:** Coefficients for calculating RAK levels for each patient group.

	**General Fluoroscopy**							
Group	Slope (m)	S_50_ (50% level)	S_90_ (90% level)	S_98_ (98% level)	Intercept $\hat{c}$	*RAK_avg_* (mGy)	Average thickness (cm)	Adult *RAK_avg_* (mGy) @ 23 cm
GI	0.204	0.465	1.133	1.603	−2.29	1.76	14.0	10.92
TP	0.171	0.752	1.835	2.596	−3.45	0.37	14.5	1.57
VCUG	0.183	0.443	1.080	1.527	−2.54	0.83	12.9	5.21
VSS	0.030	0.487	1.187	1.679	0.54	2.25	9.8	3.24

	**Fluoroscopically Guided Interventions**							
Group	Slope (m)	S_50_ (50% level)	S_90_ (90% level)	S_98_ (98% level)	Intercept $\hat{c}$	*RAK_avg_* (mGy)	Average thickness (cm)	Adult *RAK_avg_* (mGy) @ 23 cm
Low	0.052	0.87	2.12	3.00	1.15	6.03	13.4	9.6
Medium	0.074	0.93	2.28	3.23	1.63	14.8	14.9	26.4
High	0.096	0.74	1.82	2.59	3.88	154	14.5	306

	**Mobile C‐arms**							
Group RAK	Slope (m)	S_50_ (50% level)	S_90_ (90% level)	S_98_ (98% level)	Intercept $\hat{c}$	*RAK_avg_* (mGy)	Average Age (Years)	Adult *RAK_avg_* (mGy) @ 21 year‐old
Low	0.049	1.060	2.585	3.656	−0.54	0.99	10.8	1.64
Medium	0.055	0.968	2.360	3.338	0.41	2.64	10.2	4.76
High	0.092	1.159	2.830	4.010	1.01	6.87	10.0	18.86

Abdomen + MSK, abdominal or musculoskeletal system; Angio + Neuro, body or head angiograms; GI, Gastrointestinal; Pics + cleral, Pick line or Scleral Studies; RAK, air Kerma without backscatter at interventional reference point; S_50_, 50% upper prediction level; S_90_, 90% upper prediction level; S_98_, 98% upper prediction level; TP, Tube Placement; VCUG, Voiding Cystourethrogram; VSS, Video Swallow Study.

The RAK alert level based on the 98^th^ upper prediction level can then be calculated for a 2 year‐old toddler with a 12 cm thick anteroposterior projection and for the adult patient with a 25 cm anteroposterior thickness using Equation (5) and coefficient data from Table 3:

$${\hat{RAK}}_{\alpha }\left(x_{new}\right)=EXP\left(m\cdot x_{new}+s_{\alpha }+\hat{c}\right)$$

$${\mathrm{RAK}}_{12\mathrm{cm}}=EXP\left(0.204\cdot 12+1.603-2.104\right)=EXP\left(1.947\right)=7\;mGy\;\mathrm{alert}\;\mathrm{level}.$$

$${\mathrm{RAK}}_{25\mathrm{cm}}=EXP\left(0.204\cdot 25+1.603-2.104\right)=Exp\left(4.599\right)=99\;mGy\;\mathrm{alert}\;\mathrm{level}.$$

